# Characterization of a new human astrocytoma cell line SHG140: cell proliferation, cell phenotype, karyotype, STR markers and tumorigenicity analysis

**DOI:** 10.7150/jca.40802

**Published:** 2021-01-01

**Authors:** Yanyan Li, Ting Sun, Zhi Chen, YunXiang Shao, Yulun Huang, Youxin Zhou

**Affiliations:** 1 Department of Neurosurgery & Brain and Nerve Research Laboratory, The First Affiliated Hospital of Soochow University, 188 Shizi Street, Suzhou, Jiangsu, China.; 2 Department of Pathology, The First Affiliated Hospital of Soochow University, 188 Shizi Street, Suzhou, Jiangsu, China.

**Keywords:** Proliferation, karyotype, STR markers, Xenograft tumor

## Abstract

**Background:** Primary tumor Cell was an important tool for tumor research. Here, a new astrocytoma cell line SHG-140 was established and its proliferation, phenotype, karyotype, STR authentication, pathological characteristics, and characteristics of the cells' intrancranial xenografts of nude mice were studied.

**Methods:** Primary SHG-140 culture was performed in DMEM/F12 medium with 10% FBS. Cell proliferation, karyotype analysis, cell immunofluorescence and STR authentication of SHG140 cells were performed. HE staining and immunohistochemistry, Whole oncogene high flux sequencing of the patient sample were carried out. SHG140 cells were injected into the brain of nude mice, HE staining and immunohistochemistry of intracranial xenograft tumor were detected.

**Results:** Cell immunofluorescence demonstrated that SHG140 cells were positive for A2B5 (Glial precursors ganglioside), GFAP (Glial fibrillary acidic protein), Nestin, S-100 (Acid calcium bingding protein), Olig2 (Oligodendrocyte transcription factor 2) and Ki67 (Nuclear-associated antigen), cells negatively stained for Vimentin. Cell proliferation curve revealed that SHG140 proliferated slightly within 48 h, which then significantly proliferated to the fourth day. Karyotype analysis demonstrated its total number of chromosomes was 55, having trisomy of chromosome 6, 7, 8, 9 and X, and tetrad of chromosome 1 and 21, chromosomal deletion and rearrangement were observed. STR markers analysis showed the cells were derived from human male. SHG140 cells had tumorigenic properties - the intracranial injection of these cells into nude mice gave rise to growing tumors. We found that the glioma tissue was diffusively positive for GFAP, Nestin, slightly positive for Olig2, S-100; the positive rate of Ki-67 was 65% and negative for Vimentin. SHG140 cells were tumorigenic, GFAP, Nestin, S-100 Olig-2, the proliferation marker Ki-67 were expressed in its intracranial xenograft, Vimentin was negative expressed. Whole oncogene high flux sequencing of the patient tissue showed TP53, PTEN, IDH1 and PTCH1 mutation were existed.

**Conclusions:** Our study showed that SHG140 was an astrocytoma glioma continuous cell line derived from a human adult male, having a strong tumorigenicity in nude mice, which made it wound be a useful model for the study of human glioblastoma multiforme.

## Introduction

Glioblastoma Mutliforme (GBM) is the most common primary brain tumor and has a poor prognosis. The average survival rate is approximately 15 months even if the patient surgical resection combined with chemotherapy and radiotherapy 1. Cell culture is a key technology for cancer research, as it allows scientists to study the biology of tumor cells in an environment with controlled variables 2.

We previously established the first human glioma cell line SHG-44 in China 3 and a cell line SHG139 from a WHO II grade fibrous astrocytoma patient 4. To further study glioblastoma, an astrocytoma glioma continuous cell line SHG140, obtained from a human glioblastoma, maintained in culture for over 3 years and subcultured more than 50 times. Biological characteristics of the cell line, pathological and histological characteristics of intracranial tumor in nude mice related to this cell line will be elaborated in this work.

## Materials and Methods

### Tumor origin

We obtained the glioma tissue specimen from a 56-year old man suffering from local recurrence at 3 months after receiving tumor removal surgery and subsequent chemotherapy without focal radiation at the department of neurosurgery in the First Affiliated Hospital of Soochow University in 2014. This study was approved by the ethics committee of Soochow University. The tissue in glioma sample library of our laboratory was registered as Suzhou human glioma NO: 140, so was named SHG140. Nerve tumors' standard of WHO in 2007 was used for diagnosis, and fresh glioma specimen removed in operating room will be send to tissue processing room in Pathology for diagnosis. The remaining tumor specimen will be placed in DMEM/F12 medium with 1 ug/ml Amphotericin B (Sigma, A9528), and 50 ug/ml Gentamicin (FisherSci BP918-1) for cell culture.

### Primary cell culture and subculture of human glioma

In a certified biosafty hood, fresh tumor tissue in the DMEM/F12 medium was washed and minced in PBS followed by Trypsin-EDTA (0.2 mM EDTA, 0.05% Trypsin) at 37°C for 30-45 min. The isolated cells were resuspended in DMEM/F12 with 10% FBS, 1 ug/ml Amphotericin B and 50 ug/ml Gentamicin. Next, cells were seeded in a culture flask and maintained in a humidified 5% CO_2_ incubator (Thermo Fisher Scientific Inc. 3110, Waltham, MA, USA) at 37°C, with the culture medium replaced every other day. When cells reached 80% confluence, they were passaged after Trypsin-EDTA digestion. SHG140 cells were authenticated by Genetic Testing Biotechnology Corporation (Suzhou, China) using short tandem repeat (STR) markers.

### Immunofluorescence assay of SHG140

Glass microslides were coated with poly-L-lysine and single cell suspensions were dropped on glass slides. Cells were fixed with 4% paraformaldehyde at room temperature, then blocked with goat serum and permeabilized with 0.1% Triton X-100. Primary antibodies A2B5, Ki67, GFAP, Olig-2, Nestin, S-100 and Vimentin were added and incubated at 4°C overnight. Cells were rinsed with PBS, and fluorescence-labeled secondary antibodies were added and incubated at room temperature, kept off from light for 1h. Then cells were stained with DAPI, rinsed with PBS and sealed with mounting medium. The slides were analyzed by fluorescence microscopy (Olympus, Japan).

### Determination of glioma cell proliferation

1×10^3^ and 2×10^3^ cells were seeded in 96-well plates and cultured in medium with 10% fetal bovine serum. 5 time points were set in quadruplicate: 0d, 1d, 2d, 3d, 4d. At each time point, cells in every well were treated with 100ul of 3-(4, 5-dimethythiazolyl) -2, 5- diphenyltetrazolium bromide (MTT) solution (1mg/ml in DMEM/F12), incubated at 37°C for 1 h. Supernatant medium was discarded, added with 100 ul DMSO (dimethylsulfoxide) for shaking 1 h at room temperature to solubilize formazan crystals. Absorbance was measured at 560 nm wave length (reference wave length was 630 nm).

### Karyotype analysis

SHG140 monolayer cells of the 48^th^ generation in log-phase growth were harvested after mitotic arrest with colchicine (25 μg/ml) for 6 h and enzymatic dispersal with trypsin/EDTA. Preheated 0.075 mM KCl was added for 30 min at 37°C. Then, fresh stationary liquid (methanol: glacial acetic acid =3:1) was added and cells were centrifuged at 1200 rpm for 10 min. The pellets were fixed at room temperature for 10 min (4 times), and 500 μl stationary liquid was added to resuspend the cells. Afterward, cell droplets were spotted onto clean microscope glass slides that had been soaked in 4 to 6°C ice water. The samples were baked in 80°C constant temperature drying oven for 15 min. G-banding metaphase spreads images were gotten and analyzed.

### Intracranial xenograft tumors in nude mice

Twenty BALB/C female nude mice, 5-6 weeks old, were purchased from the SiLaiKe Laboratory Animal Limited Liability Corporation in Shanghai. To build the intracranial xenograft tumors, 10^6^ cells were injected into the right frontal lobe of nude mice brain, tumors began to develop after 9 weeks. Histological examination was routinely performed in serial procedures.

### HE staining and immunohistochemistry of the patient glioma tissue

The tissue from the patient was fixed in 10% formalin, dehydrated with automatic tissue hydroextractor, then embedded in paraffin. Serial 5 μm thick slides were cut with a slicer, incubated for 1h at 80°C, deparaffinized with xylene for two times, hydrated with ethanol gradient and stained with hematoxylin and eosin (H&E) (Sigma-Aldrich, USA). In the meanwhile, immunohistochemical staining was performed for GFAP, Nestin, Oligo-2, S-100, Vimentin and Ki-67 detection. Endogenous peroxidase inactivation was conducted with 3% H_2_O_2_, and antigen retrieval was performed in a microwave. Afterward, primary antibodies were added to each slide and the sections incubated with biotin-labeled secondary antibodies for 10 min. The staining were carried out using the 3, 3'-diaminobenzidine substrate (DAB) (RD, America), counterstained with hematoxylin, then analyzed by optical microscopy.

### HE staining and immunohistochemistry of mouse intracranial xenograft tumor

Nude mice with intracranial xenografts were euthanized then the xenografts were removed and embedded in paraffin. The sections were cut 5 μm thick and mounted on poly-L-lysine coated glass slides. Slides were incubated at 80°C for 1 h and deparaffinized with xylene, hydrated with gradient ethanol and stained with hematoxylin and eosin staining (H&E) (Sigma-Aldrich, USA). Slides were meanwhile stained immunohistochemically with primary antibody of GFAP, Nestin, S-100, Oligo-2, Ki-67 and Vimentin. DAB was used to stain the slides and hematoxylin was used to counterstain the nucleus. The slides were analyzed by optical microscopy.

### Whole oncogene high flux sequencing of the patient tissue

The tumor tissue from patient was sent to FIRST^TM^ imension corporation (Suchow, China), DNA was abstracted with GeneRead^TM^ DNA FFPE Kit, then purified and concentration of DNA was detected. Then, DNA was cut randomly into 250-300bp length fragment with a Covaris Crusher, two terminals of the fragments after terminal repaired and A tail added were joined to joints and carried out with pre-PCR. Customized probes were used to hybrid capture, then elution and post-PCR was processed, a DNA library was obtained. Agilent 2100 was used to determine insert size of the DNA library, then Second generation high throughput sequencing was perfumed with FD-600 production (FIRST^TM^ imension, Suzhou, China) on Illumina Hiseq platform, approximately 600 cancer gene exons was detected.

## Results

### Primary and passage culture of SHG140 cells and their molecular characteristics

**Figure 1:**
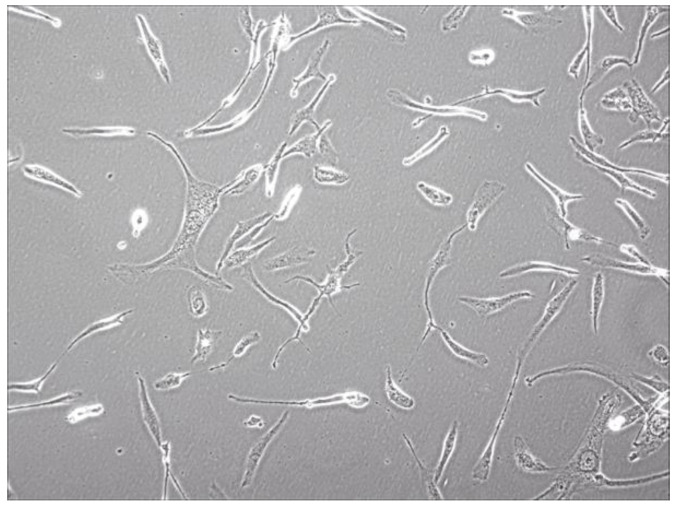
Photomicrograph of SHG140 cells in the 10^th^ generation (X400).

**Figure 2:**
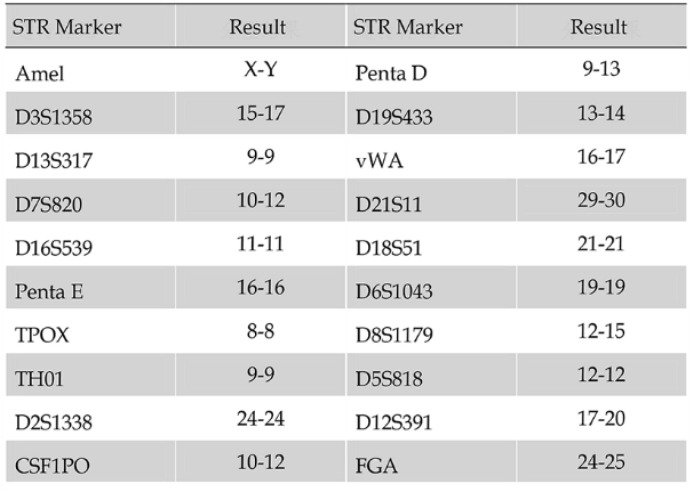
STR profile of SHG140 cells in the 33th generation.

The tissue from neurosurgery was chopped by an eye scissors in medium and then digest with 4-5 tumor volume of Trypsin-EDTA(0.2mM EDTA, 0.05 Trypsin) at 37°C for 30-45 min. Cells were span at 1000 rpm for 5 min, resuspended with DMEM/F12 medium, pipetted up and down with a pipette until smooth. A sterile cell strainer (70um Nylon) was used to filter cell suspension and collected single cells. Primary SHG140 cells were plated in a sterile flask by adding the DMED/F12 with 10% FBS after spinning. Cells were passaged and up to 10 times later, passages in monolayer format were intended to grow fast and stably. Cells were adherent in monolayer, with irregular cell morphology, mostly bipolar and fusiform, a few triangular and quadrangular, nuclear atypia was significant, with visible round and irregular polygonal shape, 1 or 2 nucleus in a cell (**Fig. 1**). STR authentication of SHG140 in the third, 13^th^ and 33^th^ generation was perfumed, the results were same, showing SHG140 was from a male glioma patient, and the STR profile was different from all cell lines' STR profile in the CCTA cell bank (**Fig. 2**).

**Figure 3:**
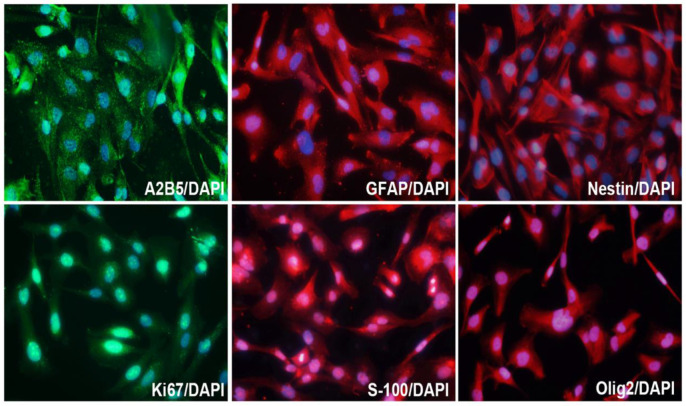
Molecular markers expression of SHG140 in the 10^th^ generation. The immunofluorescence staining of A2B5 (++), GFAP (+++), Nestin (+++), Ki67 (++), S-100 (++), Olig2 (++). DAPI is a nuclear dye used as counterstain in immunofluorescence (X400).

In order to study the characteristics of SHG140 cells, immunofluorescence staining was performed in the 10^th^ generation cells, and the expression of A2B5, GFAP, Nestin, Olig2, S-100 and Ki67 were positive (**Fig. 3**), while, the expression of Vimentin was negative (the result was not showed).

### Cell proliferation of HG140 cells

**Figure 4:**
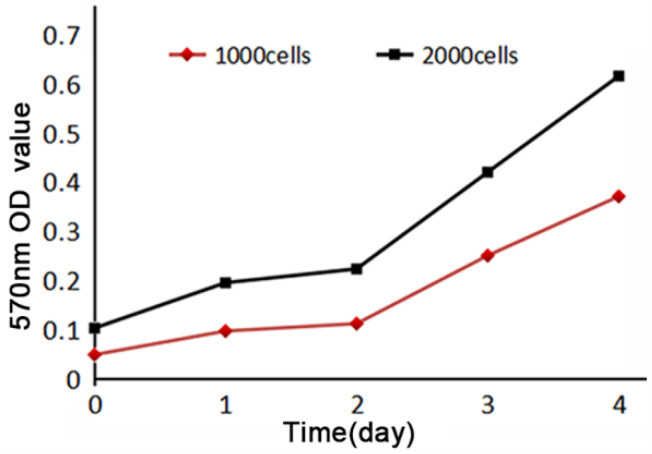
Cell proliferation curve of SHG140. Horizontal axis indicated time, vertical axis indicated the 570nm absorbance value.

MTT assay of SHG140 cells in the 31^st^ generation was performed to detect the cell proliferation, 570 nm OD value increased slightly within 48 h, then significantly after 48h. The curve of proliferation showed that SHG140 cells grew slowly within 48 h, and then cells were grown at a rapid speed (**Fig. 4**).

### Chromosome karyotype of SHG140 cells

**Figure 5:**
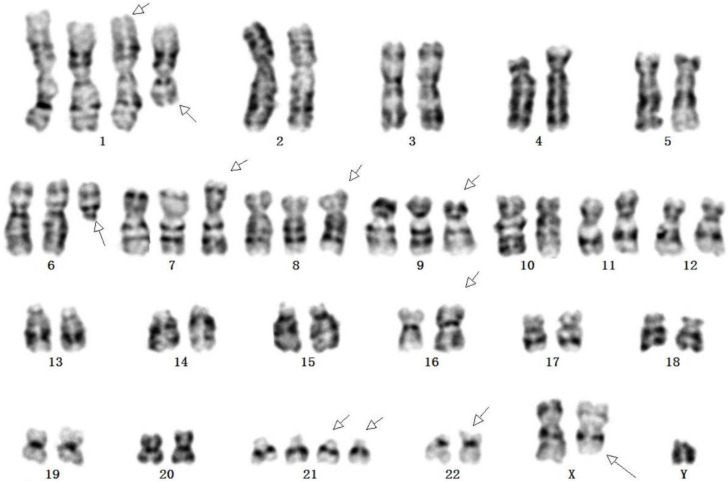
Karyotype of SHG140 cell line. Arrows indicated abnormal chromosomes.

SHG140 cells in the 48^th^ passages were harvested for G-banding karyotype analysis after digestion, fixation, and staining. G-banding metaphase images were obtained under a digital camera microscope and analyzed. Chromosome karyotype pairing showed that the total number of chromosomes was 55, Y were one copy; 2, 3, 4, 5, 10, 11, 12, 13, 14, 15, 16, 17, 18, 19, 20 and X were two copies; 6, 7, 8 and 9 were three copies; 1 and 21 were four copies. Chromosome shape was irregular distortion, the long arm of chromosome 1 and 6 were deleted, chromosomal rearrangement was found in the short arm of chromosome 16 and 22, and the long arm of chromosome X (**Fig. 5**).

### Clinical radiological and pathological data of the patient and pathological analysis and immunohistochemistry of SHG140 intracranial xenografts

**Figure 6:**
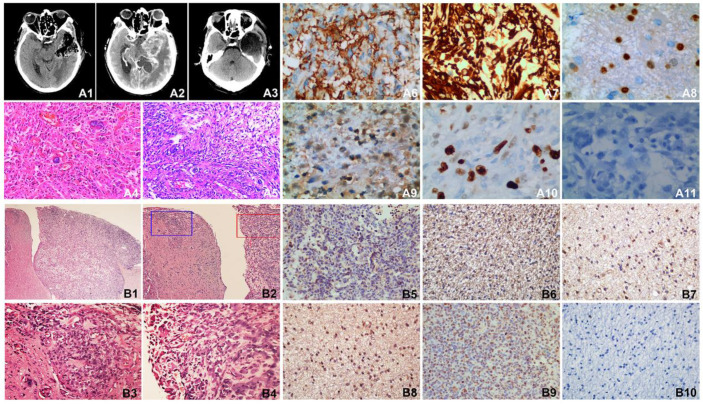
MRI of the patient, HE staining and immunohistochemisty of the patient tissue and SHG140 intracranial xenografts. A1: The first post-surgery contrast-enhanced MRI, A2: The second pre-surgery contrast-enhanced MRI, A3: The second post-surgery contrast-enhanced MRI. A4: HE staining of the first surgery, A5: HE staining of the second surgery. A6-A11: Immunohistochemical staining of GFAP, Nestin, Olig-2, S-100, Ki67 and Vimentin in patient tissue. B1: HE staining of mouse intracranial xenograft (X40), B2: HE staining of mouse intracranial xenograft, the left part of image displayed the invasive tumor of the intracranial xenograft, the right part of image displayed the intracranial xenograft(X100), B3: HE staining in the blue frame from B2 (X400), B4: HE staining in the red frame from B2 (X400). B5-B10: Immunohistochemical staining of GFAP, Nestin, Olig-2, S-100, Ki67 and Vimentin in naked-mouse intracranial xenograft.

The first post-surgery contrast-enhanced MRI showed the tumor mass on the anterior left temporo-parietal lobe was completely resected (**Fig. 6**, A1). The second pre-surgery contrast-enhanced MRI indicated the tumor recurred on the near lobe of tumor resection (**Fig. 6**, A2). The second post-surgery contrast-enhanced MRI indicated the tumor mass was completely removed again (**Fig. 6**, A3). Pathological determination after the first surgery (**Fig. 6**, A4) and the second surgery (**Fig. 6**, A5) both showed overt nuclear atypia, a few megakaryocytes, dikaryocytes and polykaryocytes, irregular nuclear hyper-chromia and hyperchromasia, no obvious mitotic count, necrosis; high tumor cell density, nucleo-cytoplasmic ratio was increased; clear and visible microvasculars, more microvasculars were existed in A5; WHO IV astrocytoma. Immunohistochemistry revealed GFAP and Nestin were diffuse positive, a few positive Olig-2 and S-100 were visible, the expression ratio of Ki-67 was approximately 65%, it is negative for Vimentin (**Fig. 6**, A6-A11).

Intracranial SHG-140 xenografts showed obvious boundary, but there was the metastasis of tumor nearby and infiltrative growth (**Fig. 6**, B1-B2); a high density of tumor cells were viewed in the primary site of xenograft and the site of metastatic xenograft, with spindle-, circular- and other irregular shape. Cells displayed overt nuclear atypia, with nuclear hyper-chromia and hyperchromasia, the site of metastatic xenograft showed infiltrative growth and no obvious boundary with the nearby tissue, abundant microvasculars were found in xenograft (**Fig. 6**, B3-B4). Immunohistochemical staining revealed there were many GFAP positive tumor cells (**Fig. 6**, B5). Nestin were also diffusely positive (**Fig. 6**, B6). A few Olig-2 and S-100 were detected (**Fig. 6**, B7-B8).The positive rate of tumor proliferation index Ki67 was approximately 60%, which is accordance with the rate of Ki67 detected in patient's tumor tissue (**Fig. 6**, B9). Vimentin was not expressed in the mouse xenograft (**Fig. 6**, B10).

### Whole oncogene high flux sequencing of the patient tissue

**Figure 7:**
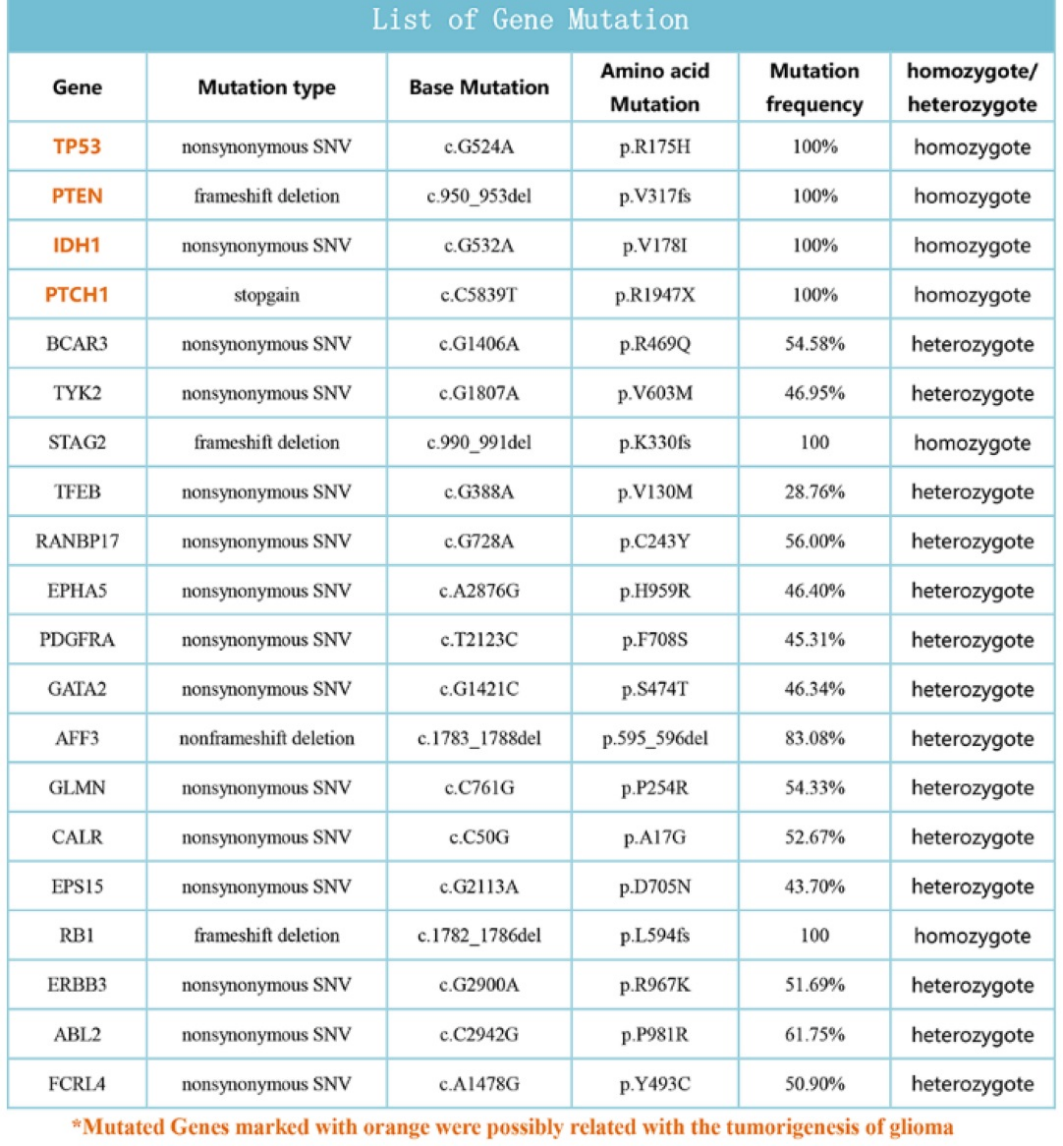
List of some mutation genes from Whole oncogene high flux sequencing of the patient tissue.

Approximately 600 cancer gene exons sequencing was perfomed according to the experiment procedure of FIRST imension biotechnology Co., Ltd. The data of sequencing was analyzed and some important gene mutations were obtained, TP53, PTEN, IDH1 and PTCH1 was possibly related with the tumorigenesis of the patient (**Fig. 7**).

## Discussion

For several decades, immortal cancer cell lines have played an important role in our research of cancer biology and the potential efficacy of anticancer drugs 5. However, accumulation of genetic aberrations of cancer cell lines often occurs with increasing passage numbers has limited their application 6,7. Therefore, it is crucial for establishing primary cancer cell lines. Glioblastoma research would benefit from culture conditions that could maintain the original phenotype of the cells 2.

Here, we reported the development and characterization of a stable cell line SHG140 of human glioblastoma multiforme from recurrence gliobastoma patient. SHG140 was obtained successfully in DMEM/F12 with 10%FBS, and passaged stably to the 50^th^ transfers. Multiple karyotypic abnormalities have been existed. Three copies existed in chromosome 6, 7, 8 and 9, four copies occurred in chromosome 1 and 21. Chromosome shape was irregular distortion, the long arm of chromosome 1 and 6 deleted, chromosomal rearrangement occured in the short arm of chromosome 16 and 22, and the long arm of chromosome X. STR profile demonstrated SHG140 cells were derived from human male. Immunofluorescence staining of SHG140 in the 10^th^ transfers showed A2B5, GFAP, Nestin, Olig-2, S-100 and Ki67 were positive expressed but Vimentin was negative expressed. Tissue immunohistochemistry from the patient and the nude mice had the similar molecular markers: A2B5 and GFAP were diffuse positive, a few Olig-2 and S-100 were positive, the expression ratio of Ki-67 was similar, Vimentin was both negative.

In a study, they reported that all human gliomas contain a pool of glial progenitor cells expressing A2B5 ganglioside recognized by the neural specific monoclonal antibody A2B5. The A2B5^+^ cells derived from human GBM show a high proliferation and are genetically transformed 8, demonstrating differentiation properties such as coexpression of GFAP. GFAP is an intermediate filament glial fibrillary acidic protein is mainly expressed in mature astrocytes. A study showed GFAP was generally strong in both low grade astrocytomas and glioblastomas. Therefore, GFAP is frequently used as a trustworthy marker of glial astrocytes and tumors in clinical and fundamental experiments 9-11. Astrocytomas, oligodendrogliomas and oligoastrocytomas were collectively reffered to as diffuse gliomas. The oligodendrocyte lineage gene Olig-2 was highly expressed in all diffuse gliomas, but higher Olig-2 in anaplastic oligodendrogliomas and GBM 12,13. Nestin, an intermediate filament protein highly expressed in high grade gliomas, such as anaplastic astrocytoma and glioblastoma. Nestin is also a marker for detecting the infiltration of malignant cells in surrounding tissue 14-16. S-100 protein family belongs to the superfamily of the EF-hand type, acting at the level of cell proliferation, cell death, cytoskeletons and cell migration. S-100 protein ubiquitously existed in glial cells, astrocytes, macroglial and microglial cells of the central nervous system. The S100B immunoreactivity was observed in many glioblastoma multiforme 17,18. SHG140 cells derived from a patient with glioblastoma, S-100 expressed in cells.

GBM-derived explants generated a large number of migrating cells moving out of these explants. These migration cells were very anarchic, giving a typical disorganized cell distribution pattern to the outgrowth cells. This phenomenon remind us of the migrated glioma cells from the tumor mass into adjacent normal brain tissue generate multiple new foci of tumors *in vivo* 19. MRI images of the patient in this study showed that GBM recured at the foci near the first resection place. HE staining of intracranial orthotopic tumor of the SHG140 cells in the nude mice demonstrated that this tumor cells invaded into the surrounded brain tissue.

Gain of chromosome 7p was a poor prognostic marker and associated with shorter survival, occurred most frequently in older patients' 20. Our chromosome karyotype of SHG140 showed the gain of chromosome 7. TP53, the tumor suppressor encoded by the TP53 gene on chromosome 17p, plays an important role in a number of cellular processes. Studies showed TP53 mutation frequencies are common in astrocytoma and contribute to astrocytoma tumorigenesis 21-24. PTEN mutation existed in many human cancers including glioma, melanoma, prostate and breast, have been reported 25-27. PTEN, as a tumor suppressor gene, can regulate the PI3K/AKT signaling pathway 25. PTEN alteration is a hallmark for progression to high malignant cancer 26. IDH1 mutation is an important prognostic hallmark of secondary glioblastomas, and is associated with the increase of overall survival 28,29. Interestingly, many glioblastomas carrying IDH1 mutations have TP53 mutations 30. Our whole oncogene sequencing result showed TP53, PTEN, IDH1 and PTCH1 existed in SHG140, and they were related with tumorigenesis of glioma.

In conclusion, SHG140 cell was an astrocytoma glioma continuous cell line derived from a patient with glioblastoma. Our new cell line will provide a good cell biochemical tool for fundamental researches of glioblastoma.

## Supplementary Material

Supplementary materials.
