# Supplementary material for: Characterization of a new human astrocytoma cell line SHG140: cell proliferation, cell phenotype, karyotype, STR markers and tumorigenicity analysis
Source: J Cancer. 2021 Jan 1;12(2):371–8. doi: 10.7150/jca.40802 (PMC7738992; doi:10.7150/jca.40802)
Supplement: Supplementary file 1 — Supplementary materials. [file jcav12p0371s1.pdf]

The ethics approval of research involving animals

医学伦理审查报告

2016伦审（申报）批第026-2号

我院脑神经研究室拟申报“基于蛋白敲除策略降解IDH1R132H突变促进胶质瘤干细胞分化的研究”科研项目，我院医学伦理委员会对该项目涉及的伦理问题进行了审查。

拟申报项目信息：

项目类型：2016年国家自然科学基金项目

研究项目名称：基于蛋白敲除策略降解IDH1R132H突变促进胶质瘤干细胞分化的研究

承担单位：苏州大学附属第一医院

项目负责人：李炎炎　　　　职称：研究实习员

研究起止日期：2017.1.1-2019.12.31

涉及动物实验的主要内容：

利用裸鼠做为实验材料，需裸鼠300只，制作颅内给药套管模型，经由给药套管注入IDH1m胶质瘤干细胞，建立肿瘤模型，然后，将合成的融合多肽经给药套管注入瘤内探索融合多肽对该模型鼠的治疗效果。

审查评议意见

经我院医学伦理委员会审议，该研究的实验设计和实施方案充分考虑了安全性和公平性原则，研究内容不构成对受试者的伤害和风险，受试者的招募将安全基于自愿和知情同意原则，并尽最大限度保护受试者的隐私。

结论

同意项目负责人申报该项研究。

苏州大学附属第一医院医学伦理委员会

2016年02月26日

The ethics approval of research involving human

苏州大学附属第一医院医学伦理委员会审批件

（2013）伦研批第（035）号

研究项目名称	候选 lncRNA 调控 A2B5+/CD133-胶质瘤干细胞干性维持和侵袭的机制研究
申报项目	国家自然科学基金
研究病例总数	20 例
申请科室	神经外科
项目负责人	周幽心

审查文件名称	
✓	伦理审查申请书
✓	临床研究方案
✓	知情同意书
✓	研究者履历表
✓	关于试验样本使用管理声明

审批意见：

根据项目负责人申请，院医学伦理委员会审议，同意申请人申报国家自然科学基金，请严格按照我国人体生物医学研究的相关规定及相关法律法规要求开展临床研究。

主任委员 [illegible]

苏州大学附属第一医院医学伦理委员会（盖章）

2013 年 02 月 26 日

伦理委员会联系热线：0512-67780081
地址：江苏省苏州市十梓街 188 号
传真：0512-67780655
邮编：215006

The patient's Informed consent

Informed Consent

关于使用组织样本开展科学研究的知情同意书

一、 组织样本在科学研究中的重要作用

1. 如果确定你可能患有肿瘤并需要做活检或外科手术，我们将取一部分组织进行必要临床检测并告知你结果，这些检测结果对于准确诊断和指导治疗十分重要。

2. 首先在征得你同意的情况下，我们会将这些剩余的组织保存起来并进一步用于科学研究，以明确肿瘤发病的原因。这些研究将会对肿瘤或其他疾病作更深入的了解并为有效治疗提供科学依据。请阅读“组织标本是如何用于科学研究的问答”CD，这些信息将有助于你对组织样本在科学研究中的意义有更多的了解。

3. 不论你是否被确诊为肿瘤，你提供的组织样本对于科学研究是有帮助的，因为这些生物样本可以进行与疾病相关的研究，以解决人类面临的健康问题。

4. 我们将严密保存获得的研究结果，研究报告可能不会告知你本人或你就诊的医生，暂时不会存入你的健康档案，这项研究也不会影响你目前的治疗。

二、需要考虑的问题

1. 我们将尊重你的选择，不论你是否同意保存剩余的组织样本，都不会影响对你的治疗。

2. 你可以现在就可以做出决定，同意保留你的组织样本并用于科学研究，但也可以随时改变你的决定，请与我们保持联系并及时告知你的想法。

3. 如果我们得到不同意使用的告知，将不再使用你的任何组织样本，但是如果已从利用组织样本的研究中获得了相关的知识，这些知识将用于人类的健康事业。

4. 将来，研究人员可能需要进一步了解有关你的健康状况的信息，主要是依据研究工作的要求，但是你的姓名、地址、电话或者其他的能够证明你身份的信息是保密的。

5. 在有些情况下，你的部分组织可能会用于家族性或遗传性疾病的研究，即使你的组织样本用于这一类研究，其结果也不会存入你的健康档案。

6. 你提供的组织样本只用于科学研究，获得的部分研究结果有可能会发展成为具有临床应用价值的检测方法，但是你目前没有利益可获得。

三、利益与风险

1. 这是一项科学研究工作，主要是推动科学和技术的进步，没有直接的经济效益或福利。

2. 组织标本是开展科学研究的重要资源，对人类认识肿瘤的发病规律，探索有效的预防和治疗方法具有不可替代的作用。

3. 对你自己而言，最大的风险就是泄漏你的健康信息，其实将这些健康信息透露给别人的机会是很小的。

4. 我们确保不会公布你的身份而且会使你的心理和社会伤害的机会降到最低。我们有预防措施以保护你的身份不被泄漏。你提供的组织样本和你的临床资料将会用一个编号表示，代表你的组织样本和临床资料。与你的姓名和编号联系的基本项目将以电子档案的形式保存到计算机中，只有授权人才可以看到你的相关信息，研究人员和其他采样及收集临床资料的工作人员都不会知道你的身份。

5. 我们有责任保护你的隐私，但是有一些不可预测的因素有可能导致部分信息被某些人用于识别你的身份，一般说来这种可能性是非常小的。

6. 如果你的身份和相关信息被泄漏，有可能会给你和你的家人带来烦恼。另外，与疾病相关的基因检测报告有可能影响你的健康保险和一些与身体条件相关的工作聘任。虽然这方面有法律保护，但不能完全保护人们免受歧视。

7. 由于你与你的子女、父母、兄弟姐妹和其他家庭成员的遗传学特征相似，这些风险性对他们来说同样存在，但与你相比要小得多。

四、根据你的考虑做出选择

请你仔细阅读下面各项并做出选择，在同意和不同意处标注。不论你作何种选择都不会影响你的治疗。如果有什么疑问请与你的医生和护士咨询或与我们的伦理委员会联系。我们将尊重你的选择，不论你是否同意保存剩余的组织样本，都不会影响对你的治疗。

我的组织样本可以用于肿瘤预防和诊治等方面的科学研究。

✓A 同意　　　　　　B 不同意

我的组织样本可以用于其他危害健康的疾病的科学研究。

✓A 同意　　　　　　B 不同意

我的临床资料信息可以与我提供的组织标本相联系。

✓A 同意　　　　　　B 不同意

回答上述问题后请你签名。

患者签名：秉刚　　　　　　日期：2014.7.10

医生/护士签名：周[illegible]心　　　　日期：2014.7.10
